# Randomized controlled trial of a group intervention combining self-hypnosis and self-care: secondary results on self-esteem, emotional distress and regulation, and mindfulness in post-treatment cancer patients

**DOI:** 10.1007/s11136-020-02655-7

**Published:** 2020-10-06

**Authors:** C. Grégoire, M.-E. Faymonville, A. Vanhaudenhuyse, G. Jerusalem, S. Willems, I. Bragard

**Affiliations:** 1 Faculty of Psychology, Speech Therapy and Educational Sciences, and Sensation and Perception Research Group, GIGA Consciousness, University of Liège, Liège, Belgium; 2 Interdisciplinary Algology Centre, CHU Liège, and Sensation and Perception Research Group, GIGA Consciousness, University of Liège, Liège, Belgium; 3 Medical Oncology Department, CHU Liège and University of Liège, Liège, Belgium; 4 Faculty of Psychology, Speech Therapy and Educational Sciences, University of Liège, Liège, Belgium; 5 Haute Ecole Libre Mosane (HELMo), Liège, Belgium

**Keywords:** Cancer, Emotional distress, Mindfulness, Self-esteem, Hypnosis, Oncology

## Abstract

**Purpose:**

Cancer patients often report low self-esteem and high emotional distress. Two factors seem particularly linked to these symptoms: emotion regulation strategies and mindfulness. The interest of hypnosis and self-care to relieve these symptoms is not well documented. Our randomized controlled trial aimed at assessing the effect of a group intervention combining self-hypnosis and self-care on self-esteem, emotional distress, emotion regulation, and mindfulness abilities of post-treatment cancer patients, as well as investigating the links between these variables.

**Methods:**

One hundred and four patients who had suffered from cancer were randomized into the intervention group (*N* = 52) and the wait-list control group (*N* = 52). They had to answer questionnaires before (T1) and after the intervention (T2). Nine men were excluded from the analyses, leading to a final sample of 95 women with cancer. Group-by-time changes were assessed with MANOVA, and associations with self-esteem and emotional distress were investigated with hierarchical linear regression models.

**Results:**

Participants in the intervention group (mean age = 51.65; SD = 12.54) reported better self-esteem, lower emotional distress, a decreased use of maladaptive emotion regulation strategies, and more mindfulness abilities after the intervention, compared to the WLCG. This increase in mindfulness explained 33% of the improvement of self-esteem and 41.6% of the decrease of emotional distress in the intervention group. Self-esteem and emotional distress also predicted each other.

**Conclusion:**

Our study showed the efficacy of our hypnosis-based intervention to improve all the investigated variables. Mindfulness predicted the improvement of self-esteem and emotional distress. The primary impact of our intervention on mindfulness abilities seems to explain, at least in part, its efficacy.

**Registration:** ClinicalTrials.gov (NCT03144154). Retrospectively registered on the 1st of May, 2017.

## Background

Cancer and its treatment have different negative consequences on patients. First, they often report a *low* *self-esteem*, linked to treatments’ physical, social, and psychological side effects [1–3]. There is no consensus for the definition of self-esteem, but it can be described as the “evaluative component of a broader representation of self” [4] or the appreciation, feeling, and consideration that one has about oneself [5]. Low self-esteem is known to be associated with lower quality of life, and lower life and social satisfaction [3, 6, 7]. Another frequent symptom is *emotional distress* [8–12], which is known to last sometimes for years after treatments [9] and which negatively impacts treatment adherence and results, as well as patients’ general quality of life [8, 13, 14]. In the general population, these two variables are known to be negatively correlated, but the direction of the causality is not clear: it is not known if a low self-esteem induces higher emotional distress, or if emotional distress impacts self-esteem [2, 15–17].

However, *emotional regulation strategies* are known to mediate the relationship between self-esteem and emotional distress [15, 16]. They are also known to correlate with emotional distress [18, 19]. Emotional regulation strategies can be defined as the “extrinsic and intrinsic processes responsible for monitoring, evaluating, and modifying emotional reactions, especially their intensive and temporal features, to accomplish one’s goal” [20]. Another variable which has been recurrently associated with lower emotional distress as well as other quality-of-life-related variables (e.g., coping, emotional regulation) is *mindfulness* [21, 22]. Mindfulness refers to the “individual’s characteristic tendency to maintain awareness of the present moment in a non-reactive and non-judgmental manner” [21]. It is a distinct concept from mindfulness practice, which involves “deliberately engaging in mindful exercises to foster a state of mindfulness” [21]. Thus, mindfulness can be conceptualized as of a state of mind, while mindfulness practice is a volitional behavior. Only mindfulness (and not mindfulness practice) will be investigated in this paper.

**Fig. 1:**
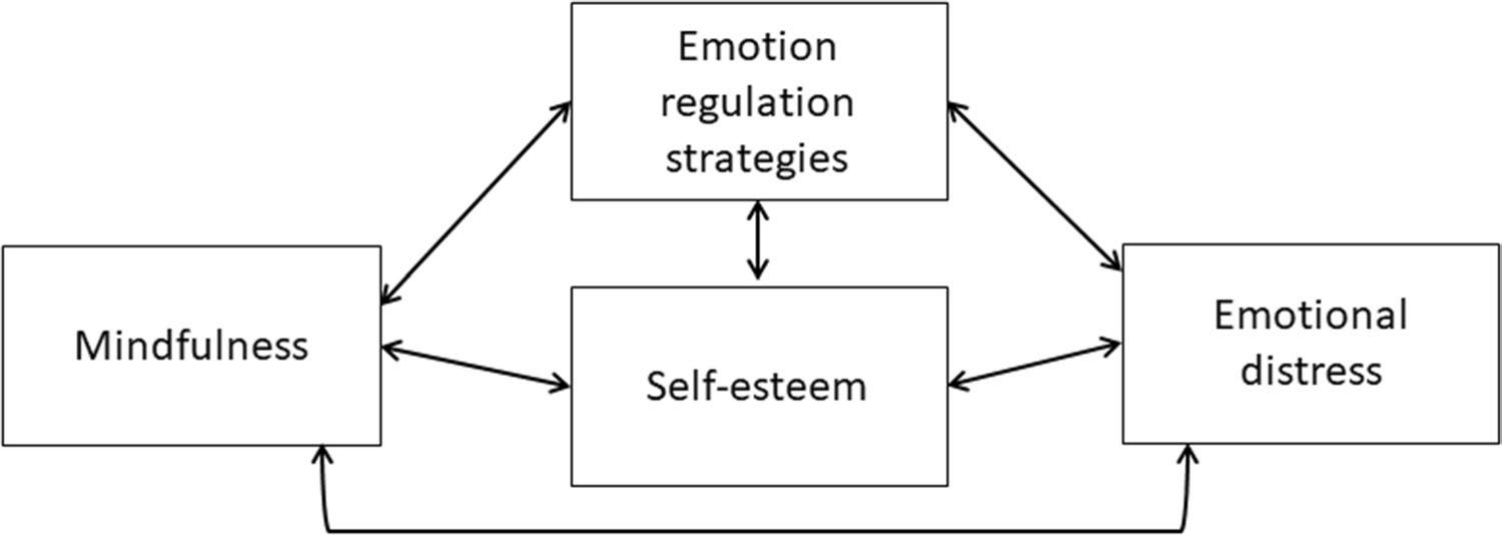
Summary of the links between the investigated variables

The correlation between mindfulness and emotional distress could be mediated by different other factors, such as, for example, self-esteem and emotion regulation [23–25]. Thus, self-esteem, emotional distress, emotion regulation strategies and mindfulness seem to be linked by strong but complex relationships, not all of which are fully understood (see Fig. 1).

Some psychological interventions have been tested to improve self-esteem and emotional distress of cancer patients [26–29]. In oncology settings, there is a growing interest in alternative methods in order to manage treatment side effects in a non-pharmacological way. Indeed, complementary and alternative medicine, such as hypnosis, is estimated to be used by 43% of patients with cancer [30, 31], and is becoming popular because of its ease of integration into cancer care [32]. Hypnosis is characterized by three components: absorption (in an imaginative experience), dissociation (from the environment), and suggestibility (to the therapist’s suggestions) [33, 34]. Some studies demonstrated the positive impact of hypnosis on cancer patients’ emotional distress, whether taught alone or combined with cognitive-behavioral or self-care techniques [29, 35], as well as first results on cancer patients’ self-esteem [36]. However, most studies focussed on breast cancer patients and suffer from some methodological pitfalls such as a lack of randomization or small sample sizes. In addition, few studies investigated the use of hypnosis to improve self-esteem in oncology, and most of them are case reports. Thus, there is a need for rigorous studies investigating the effect of hypnosis on self-esteem and emotional distress of cancer patients.

Given the strong connections between mindfulness and (1) self-esteem and (2) emotional distress, and the potential impact of hypnosis on these two variables, it seems relevant to investigate the effects of hypnosis on mindfulness as well. Even if hypnosis is different from mindfulness (in terms of goal, reality orientation, cerebral connectivity and mechanism of change, for examples. For more details, see Table 1 in [37]), both techniques have some components in common (enhanced imagery, focused and selective attention) [37] and are sometimes combined to improve self-care and well-being [38]. Moreover, van der Velden highlighted the relevance of assessing the impact of different psychological interventions, originally aimed at improving quality of life, on mindfulness abilities [39]. The intervention tested in this study combined self-hypnosis and self-care learning which, in some aspects, are related to mindfulness-based approaches (see “Intervention” section). If we confirm the impact of our intervention on our variables, and the links between them, this could deepen the understanding of the mechanisms of action of our intervention.

## Objectives

This paper focusses on secondary analyses of a randomized controlled trial the primary aim of which was to assess the efficacy of our group intervention to improve fatigue and associated symptoms in post-treatment cancer patients and to explore the predictors of the evolution of fatigue. These primary results have already been published [40].

Our paper’s primary aim is to assess the efficacy of a group intervention combining self-hypnosis and self-care learning to improve self-esteem, emotional distress, emotional regulation, and mindfulness of post-treatment cancer patients. Our secondary aim is to investigate the predictive relationships between these variables, in order to identify which ones predict the evolution of self-esteem, and which ones predict the evolution of emotional distress.

## Methods and design

We published the protocol of this study [41] with detailed information about the design, recruitment and randomisation procedures, sample size calculation, assessments, and intervention. Therefore, we will only summarize these aspects here.

### Design

We used a longitudinal randomized wait-list controlled trial design with an intention-to-treat (ITT) analytic strategy. Excluding participants who dropped out the study from the analyses could lead to biased results because it compromises the balance created by randomization [42]. To deal with missing values due to dropouts, we used the *last observation carried forward* method [43] in which participants’ missing data are replaced by the value they obtained in the previous measurement time. Participants were randomized into two groups: the first group received immediate intervention (intervention group) and the second group received it (at the latest) 4 months later (wait-list control group; WLCG).

### Participants

Patients were mainly recruited in the University Hospital of Liège (November 2016–March 2019). The inclusion criteria were to be at least 18 years old, to be fluent in French, to present a non-metastatic invasive cancer, to have completed active treatments since less than a year, and to experience emotional or physical difficulties as established by a score of at least 4/10 on 1 of the 6 chosen items of the Edmonton Symptom Evaluation Scale [44] (physical fatigue, moral fatigue, depression, anxiety, fear of recurrence, ruminations).

### Intervention

The intervention included eight weekly 2-h sessions in groups of 8–10 participants (1st group in April 2017, last group in September 2019). They have been developed and were led by one of the authors (MEF) [45]. Participants had to complete different self-care tasks and reflexions at home between sessions based on, for example, the reinforcement of self-esteem, the enhancement of moment-to-moment awareness, the reengagement in enjoyable activities, the management of ruminations, etc. (see Table 1 for the content of each session). Self-care was also discussed in group during the sessions in order to benefit from experience sharing. Even if not directly based on mindfulness, these tasks are in line with it, as they foster an adaptive, non-judgmental, and accepting stance towards experiences. In addition, they promote the engagement in activities consistent with one’s values, needs, and interests. A 15-min hypnosis exercise was led by the therapist at the end of each session. At-home practice was encouraged, as it allows participants to learn to induce self-hypnosis, in order to take full advantage of hypnosis without the need of being guided by a therapist. It is expected that the practice of self-hypnosis will influence cognition and emotional regulation and therefore facilitate the completion of the assigned tasks [29, 46]. In this way, self-hypnosis is complementary to self-care tasks.

**Table 1:** Content of the group sessions

Session 1	Explanation: what is hypnosis Common beliefs about hypnosis Answers to participants’ questions Discussion about their choice to participate Importance of pleasing ourselves everyday Definition of 3 realistic goals to be achieved in 6 months List of personal needs Mental imagery exercises
Session 2	Reflection on personal qualities and resources and the importance of knowing them Distancing from symptoms (“I have” not “I am”) Listening to pleasant music Discussion of the way to set priorities in life (importance and urgency) Paying attention to small successes Hypnosis exercise: fluffy white cloud
Session 3	Discussion of the way we talk to ourselves and self-esteem, the importance of congratulating ourselves Doing one thing at a time Accepting to receive a “no” Importance of the coherence between our acts and our words Identification of a safe haven Hypnosis exercise: safe haven
Session 4	Reflexion about our control over difficult situations Discussion of the balance between the energy put in to a task and the gained benefit Discussion of the ideal parent/spouse/child/colleague… Learning to delegate Assertiveness: being able to say “no” Hypnosis exercise: pain and colors
Session 5	Paying attention to the gifts of life Discussion of social roles and the way we respond to others’ needs Taking time for ourselves and doing enjoyable activities Importance of physical activity Psycho-education about sleep Hypnosis exercise: levitation
Session 6	Discussion of personal resources which help to combine work and private life Discussion of the adequacy between professional activities and personal needs Finding an object that will be associated with a “Stop!” injunction, to use when we feel stressed and ruminate Discussion of the importance of being surrounded by positive people Discussion of ruminations and how to cope with them Hypnosis exercises: light journey
Session 7	Assertiveness: how to say “no” or postpone our decision Assertiveness: how to formulate a demand and chose the right moment Importance of taking quality time for ourselves instead of always being there for other people Discussion of the difficulties and constraints encountered in daily life Hypnosis exercise: dreamland
Session 8	Discussion of irritating situations and how to cope with them in a more positive way Importance of enjoying the present moment Review of the goals determined at the beginning of the sessions: have they been achieved? The importance of being proud of ourselves, to congratulate ourselves Discussion of new objectives Hypnosis exercise: stories and metaphors

### Assessments

Assessments had been conducted at two different times, 3–4 months apart (pre- (T1) and post- (T2) intervention; March 2017 to July 2019), with questionnaires.

#### Sociodemographic and medical data

Gender, age, education level, employment status, marital situation, and number of children were noted, as well as the type of cancer, time since diagnosis, cancer treatments received, and consumption of psychotropic.

#### Rosenberg’s self-esteem scale (RSES) [47, 48]

Measures global self-esteem through positive and negative feelings about the self. Scores range between 10 and 40, with scores below 31 suggesting a low self-esteem.

#### Hospital anxiety and depression scale (HADS) [49]

Measures anxiety and depression. Cut-offs scores for each dimension are 7/21.

#### Five facets mindfulness questionnaire (FFMQ) [50]

Measures five components of mindfulness: observing (noticing internal and external experiences, such as sensations, cognitions or emotions), describing (labeling internal experiences with words), acting with awareness (attending to activities of the moment, in contrast with behaving mechanically), non-judging of inner experience (non-evaluative stance towards thoughts and feelings), and non-reactivity to inner experience (allowing thoughts and feelings to come and go, without getting carried away by them). A total score is also calculated (Mindfulness), which will be used in this paper.

#### Cognitive emotion regulation questionnaire (CERQ) [51]

Investigates the cognitive emotion regulation strategies used after experiencing negative events. Nine strategies (scores ranging from 4 to 20 for each) can be categorized in two categories, which will be used in this paper: maladaptive regulation (self-blame, blaming others, rumination, and catastrophizing) and adaptive regulation (acceptance, refocus on planning, positive refocusing, positive reappraisal, and putting into perspective).

### Data analyses

All statistical analyses were performed using Statistica 13.3 (TIBCO Software Inc.) and SPSS Statistics 25 (IBM). Group-by-time changes in all the variables were processed using multivariate analysis of variance (MANOVA) with repeated measures, followed by post hoc comparisons (Tukey’s HSD test). Effect sizes for standardized differences in means between times of evaluation were calculated using Cohen’s d, with interpretation as follows: “small” (< 0.20–0.50), “medium” (0.50–0.80), and “large” effect sizes (> 0.80) [52]. In order to investigate the factors associated with the evolution of self-esteem and emotional distress, we first calculated the pre-post-intervention difference for all variables (Δ), to be used as variables in the following analyses. We then conducted four multivariable hierarchical linear regression analyses on these differences, controlling for age and time since diagnosis. Two blocks of potential predictors were entered in the analyses: the first one composed of age and time since diagnosis, and the second one composed of all the other outcomes considered in this study (emotional distress (HADS Total score), self-esteem (RSES), mindfulness (FFMQ Total score), maladaptive, and adaptive emotion regulation (CERQ), added subsequently in the model). All tests were two-tailed and the results were considered to be significant at *p* < 0.05.

## Results

### Recruitment

Of the 114 patients initially included, 10 dropped out before T1, leading to a total sample of 104 cancer patients. Twelve of them dropped out (*N* intervention group = 8; *N* WLCG = 4) between T1 and T2. Their T2 data were replaced by their data obtained during T1, according to the ITT approach. Participants were randomized into two groups (*N* intervention group = 52; *N* WLCG = 52). As there were only 9 men in the total sample, we decided to remove them from the analyses, leading to a final sample of 95 women (*N* intervention group = 48; *N* WLCG = 47). Indeed, if we had considered men in our analyses, it would have been difficult to conclude about the impact of the intervention on them, and the sample would not have been homogenous.

### Description of the sample

Table 2 displays the demographics and medical data for the whole sample and the two groups. Women in our study were on average 53.85 ± 11.91 years old. 75 of them had breast cancer (78.94%) and the second most represented cancer was digestive cancer (*N* = 5; 5.26%).

**Table 2:** Baseline participants’ demographics and medical data in each group

	Total sample (*N* = 95)	Intervention group (*N* = 48)	WLCG (*N* = 47)
Demographics			
Age (years)			
Mean (SD)	53.85 (11.91)	51.65 (12.54)	56.11 (10.90)
Range	24–78	24–78	30–78
Gender, *N* (%)			
Women	95 (100)	48 (100)	47 (100)
Marital status, *N* (%)			
Single	6 (6.32)	3 (6.25)	3 (6.38)
Married/living with partner	63 (66.32)	35 (72.92)	28 (59.57)
Divorced/separated/widowed	15 (15.79)	5 (10.42)	10 (21.28)
In a relationship but not living together	11 (11.58)	5 (10.42)	6 (12.77)
Cultural origin, *N* (%)			
Western Europe	92 (96.84)	45 (93.75)	47 (100)
Eastern Europe	3 (3.16)	3 (6.25)	0 (0.00)
Education level, *N* (%)			
Elementary school or less	1 (1.05)	0 (0.00)	1 (2.13)
Lower secondary school	8 (8.42)	3 (6.25)	5 (10.64)
Upper secondary school	25 (26.32)	14 (29.17)	11 (23.40)
Bachelor’s degree	38 (40.00)	19 (39.58)	19 (40.43)
Master’s degree	20 (21.05)	11 (22.92)	9 (19.15)
Post-graduate	3 (3.16)	1 (2.08)	2 (4.26)
Employment status, *N* (%)			
Employed full time	7 (7.37)	4 (8.33)	3 (6.38)
Employed part time	21 (22.11)	9 (18.75)	12 (25.53)
Incapacity of work/invalidity	37 (38.95)	22 (45.83)	15 (31.91)
Unemployed/student/housewife/house-husband/retired/other	30 (31.58)	13 (27.08)	17 (36.17)
Children, *N* (%)			
Yes	82 (86.32)	40 (83.33)	42 (89.36)
No	13 (13.68)	8 (16.67)	5 (10.64)
Patient medical history			
Cancer diagnosis, *N* (%)			
Breast cancer	75 (78.94)	38 (79.17)	37 (78.72)
Others	20 (21.06)	10 (20.83)	10 (21.28)
Digestive (stomach, peritoneum, pancreas)	5 (5.26)	3 (6.25)	2 (4.26)
Hematological cancer (lymphoma, leukemia)	4 (4.22)	3 (6.25)	1 (2.13)
Gynecological cancer (cervix, ovaries)	4 (4.22)	3 (6.25)	1 (2.13)
Skin	2 (2.11)	0 (0.00)	2 (4.26)
Thyroid	2 (2.11)	0 (0.00)	2 (4.26)
Ear/nose/throat	1 (1.05)	0 (0.00)	1 (2.13)
Lung	1 (1.05)	1 (2.08)	0 (0.00)
Brain	1 (1.05)	0 (0.00)	1 (2.13)
Time since diagnosis (months)			
Mean (SD)	10.65 (8.69)	9.94 (5.13)	11.38 (11.25)
Range	1–72	2–24	1–72
Surgery, *N* (%)			
Yes	92 (96.84)	46 (95.83)	46 (97.87)
No	3 (3.16)	2 (4.17)	1 (2.13)
Chemotherapy (CT), *N* (%)			
Yes	52 (54.74)	29 (60.42)	23 (48.94)
No	43 (45.26)	19 (39.58)	24 (51.06)
Radiation therapy (RT), *N* (%)			
Yes	70 (73.68)	35 (72.92)	35 (74.47)
No	25 (26.32)	13 (27.08)	12 (25.53)
Hormonal therapy (HT), *N* (%)			
Yes	60 (63.16)	30 (62.50)	30 (63.83)
No	35 (36.84)	18 (37.50)	17 (36.17)
Other treatment, *N* (%)			
Yes	15 (15.79)	9 (18.75)	6 (12.77)
No	80 (84.21)	39 (81.25)	41 (87.23)
History of cancer, *N* (%)			
Yes	18 (18.95)	8 (16.67)	10 (21.28)
No	77 (81.05)	40 (83.33)	37 (78.72)
Other chronic health problem, *N* (%)			
Yes	55 (57.89)	27 (56.25)	28 (59.57)
No	40 (42.11)	21 (43.75)	19 (40.43)
Consumption of psychotropic during the study, *N* (%)			
Yes	50 (52.63)	27 (56.25)	23 (48.94)
No	45 (47.37)	21 (43.75)	24 (51.06)

### Impact of the intervention on patients’ variables

The MANOVA revealed a significant effect of time (F(6,87) = 7.79; *p* < .001), as well as a significant group-by-time effect (F(6,87) = 3.06; *p* = .009). Post hoc comparisons revealed an improvement of *self-esteem* after the intervention (*p* < .001; *d* = 0.46), as well as a decrease of *emotional distress* (HADS: anxiety: *p* < .001; depression: *p* < .001, with effects sizes of 0.67 and 0.71 , respectively). *Emotion regulation* also improved in the intervention group (CERQ: maladaptive emotion regulation: *p* < .001, *d* = 0.67; adaptive emotion regulation: *p* = .010, *d* = 0.42). Finally, *mindfulness* (FFMQ) also increased after the intervention (*p* < .001), with a large effect size of 0.90). No significant effect was revealed in the WLCG. Table 3 displays the results for each group.

**Table 3:** Impact of the intervention on participants’ self-esteem, emotional distress, emotion regulation, and mindfulness

	T1 Intervention group (*N* = 48)	T2 Intervention group (*N* = 48)	Effect size	Evolution (T1–T2)	T1 WLCG (*N* = 47)	T2 WLCG (*N* = 47)	Effect size	Evolution (T1–T2)
	Mean (SD)	Mean (SD)	Cohen’s d	*p*	Mean (SD)	Mean (SD)	Cohen’s d	*p*
Rosenberg’s self-esteem scale (RSES)	29.63 (6.29)	31.83 (6.30)	0.46	< .001	30.02 (5.14)	30.46 (5.13)	0.16	.857
Hospital anxiety and depression scale (HADS)								
Anxiety	10.52 (4.19)	8.10 (4.38)	0.67	< .001	9.98 (4.32)	9.17 (3.97)	0.28	.341
Depression	7.17 (4.23)	4.96 (4.19)	0.71	< .001	6.72 (4.10)	6.38 (4.63)	0.14	.848
Cognitive emotion regulation questionnaire (CERQ)								
Maladaptive emotion regulation	33.84 (9.23)	29.29 (8.27)	0.67	< .001	31.11 (8.43)	30.02 (8.65)	0.18	.665
Adaptive emotion regulation	70.40 (14.75)	75.63 (14.85)	0.42	.010	71.60 (11.76)	73.83 (13.83)	0.21	.523
Five facet mindfulness questionnaire (FFMQ)								
Total score: mindfulness	118.50 (19.81)	129.15 (20.46)	0.90	< .001	117.25 (19.93)	119.43 (21.57)	0.26	.558

### Predictors of the evolution of self-esteem and emotional distress

Our secondary aim was to understand which variables impact the evolution of self-esteem (ΔSE) and emotional distress (ΔED) in each group (intervention vs WLCG). Results are displayed in Tables 4 (for self-esteem) and 5 (for emotional distress).

**Table 4:** Predictors of the increase of self-esteem (Δ SE)

Groups	Predictors	*β*	SE	*p*	*R*^2^	Adj. *R*^2^	Δ*R*^2^	Δ*F*
Intervention group (*N* = 48)	Age and time since diagnosis	.060	1.19	.619	.021	− .022	.021	.484
		.019						
	Δ Mindfulness	.839	.980	.000	.351	.307	.330	22.36
	Δ ED	.402	.917	.010	.445	.393	.094	7.25
WLCG (*N* = 47)	Age and time since diagnosis	− .223	.687	.089	.107	.065	.107	2.57
		− .211						

*Δ Mindfulness* variation of mindfulness between T1 and T2, *Δ ED* variation of emotional distress between T1 and T2. *ΔR*^*2*^ variation of *R*^2^ after adding another predictor, *ΔF* difference between hierarchical models

**Table 5:** Predictors of the decrease of emotional distress (Δ ED)

Groups	Predictors	*β*	SE	*p*	*R*^2^	Adj. *R*^2^	Δ*R*^2^	Δ*F*
Intervention group (*N* = 48)	Age and	− .039	1.17	.917	.004	− .040	.004	.086
	Time since diagnosis	.071						
	Δ Mindfulness	− .750	.900	.000	.420	.380	.416	31.54
	Δ SE	.370	.843	.010	.503	.457	.084	7.25
	Δ AER	− .282	.793	.014	.570	.519	.067	6.55
WLCG (*N* = 47)	Age and	.197	.706	.155	.083	.041	.083	1.95
	Time since diagnosis	− 1.79						
	Δ Mindfulness	− .315	.675	.031	.180	.121	.097	4.96

*Δ Mindfulness* variation of mindfulness between T1 and T2, *Δ SE* variation of self-esteem between T1 and T2, *Δ AER* variation of adaptive emotion regulation between T1 and T2, *ΔR*^*2*^ variation of *R*^2^ after adding another predictor, *ΔF* difference between hierarchical models

Concerning *self-esteem*, variations of mindfulness (Δ Mindfulness) and emotional distress (Δ ED) respectively explained 33% (*p* < .001) and 9.4% (*p* = .010) of the variance of Δ SE in the intervention group, meaning that an evolution of these two variables significantly predicts the increase of self-esteem. In the control group, we did not find any significant predictors of the increase of self-esteem. Concerning *emotional distress*, 3 factors explained 57% of the variance of Δ ED in our intervention group: variation of mindfulness (Δ Mindfulness), variation in self-esteem (Δ SE), and variation in adaptive emotion regulation strategies (Δ AER) respectively explained 41.6% (*p* = .000), 8.4% (*p* = .010) , and 6.7% (*p* = .014) of the variance of Δ ED. This means that evolutions of mindfulness, self-esteem , and adaptive emotion regulation strategies predict, at least in part, the decrease of emotional distress. In the control group, Δ Mindfulness predicted 9.7% of the variance of Δ ED (*p* = .031). All other variables not included in the tables did not significantly predict the evolution of self-esteem (non-significant factors: Δ AER and Δ maladaptive adaptation regulation (MAER)) or emotional distress (non-significant factor: Δ MAER).

## Conclusions

First, our study showed the positive impact of a group intervention combining self-care and self-hypnosis on self-esteem and emotional distress. This is in line with other studies showing the impact of psychological interventions [26–29] and hypnosis [29, 35, 36] on these variables. Our intervention also allowed positive change in emotion regulation strategies. As emotion regulation strategies are directly discussed and addressed during the sessions, these results were expected. They are in line with other studies showing the impact of psychological interventions on emotion regulation skills across different populations [53–56]. Finally, our intervention also improved the participants’ level of mindfulness. Thus, it seems that our intervention, even if not directly based on mindfulness exercises, can improve this ability, as suggested by van der Velden [39]. Indeed, the intervention sessions encouraged participants to take time for themselves, to think about themselves, about what is important to them, and about how to implement more self-care in their daily lives. The engagement in meaningful activities, the refocusing on positive experiences and the management of negative experiences in a non-judgmental way were also addressed. Self-hypnosis was seen as a facilitator for these changes. It is then understandable that our intervention led to an improvement in mindfulness, which is characterized by a present moment aware, non-judgmental, and non-reactive stance [57]. To our knowledge, our study is the first one to attest the impact of an intervention combining self-care and self-hypnosis on mindfulness. This suggests that self-hypnosis and self-care, possibly through shared components with mindfulness, can improve this ability. Future research should deepen the study of these relationships.

Our study also underlined the major role of the increase of mindfulness after the intervention in the improvement of self-esteem and emotional distress of our participants. Increase of mindfulness was also a predictor of the decrease of emotional distress in the WLCG, even if quite much smaller (9.7% in the WLCG vs 41% in the intervention group). It is possible that mindfulness improves naturally after cancer diagnosis and treatments, possibly because patients had to focus on their health and their body during cancer, but our intervention seems to reinforce this increase. Our results thus suggest that the efficacy of our group intervention to improve self-esteem and emotional distress is in part due to its ability to affect mindfulness in the first place. Different underlying mindfulness-linked processes could be involved, including increased moment-to-moment awareness, acceptance, and self-regulation, which induce a decrease of automatic, mindless, and judgmental thinking and reactivity in response to stressors [24, 25, 58]. Our results are in line with past studies showing the links between mindfulness and self-esteem [23, 59], and between mindfulness and emotional distress [21, 22, 58] (see Fig. 1). In addition, the evolution of emotional distress significantly predicted the evolution of self-esteem, while self-esteem and adaptive emotion regulation strategies were significant predictors of the decrease of emotional distress. Thus, it seems that self-esteem and emotional distress predict each other, and that our intervention impacted both of them. This is in line with numerous studies showing the negative predictive relationship between self-esteem and emotional distress, with an unclear direction [2, 15–17] (see Fig. 1). We can confirm this correlation in our study (*r*_s_ = 0.32, *p* = .002; not displayed), and the mutual influence they have on each other. However, we cannot decide on the direction of the causality.

Our study has several limitations. First, the disproportion between breast cancers and other cancers, and between men and women in our sample was not expected, as we tried to target a lot of different patients during our recruitment process. However, as the most frequent cancer in women is breast cancer, it is understandable that most women in our sample had breast cancer. Moreover men are in general less interested in psychological interventions and rarely use them compared to women [60]. In addition, other late measurement times could have been useful to investigate the long-term evolution of our data and the best moment to propose the intervention to cancer patients. A third and a fourth measurement times have been conducted (3 and 12 months post intervention) and will lead to further analyses in the future.

In conclusion, this intervention combining self-care and self-hypnosis improved cancer patients’ self-esteem, emotional distress, emotion regulation strategies, and mindfulness abilities. It seems that the positive effect of our intervention on self-esteem and emotional distress is linked to an improvement of mindfulness. Thus, it seems particularly important to increase participants’ mindfulness abilities, as it seems to be an underlying mechanism implied in their self-esteem and emotional distress improvements.

Our results lead to several scientific perspectives. First, as stated above, investigating the long-term effects of the intervention seems to be capital. Then, it would be useful to rethink the recruitment process to foster the inclusion of men and of more cancers other than breast cancer in the sample. To do this, adapting the group intervention to men’s needs seems to be important, for example by adding psycho-education or fitness components [60]. Finally, deepening the understanding of the links between self-esteem and emotional distress could be of great interest.

## Data Availability

The full protocol and dataset of this study is available upon request. Please contact the corresponding author (ch.gregoire@chuliege.be).

## References

[1] Hilger C, Schostak M, Neubauer S, Magheli A, Fydrich T, Burkert S, Kendel F (2019). The importance of sexuality, changes in erectile functioning and its association with self-esteem in men with localized prostate cancer: Data from an observational study. BMC Urology.

[2] Vidthya S, Sherina MS, Rampal L, Fadhilah SI, Ummavathy P (2019). Self-esteem among cancer patients receiving chemotherapy in selected government state hospitals, Peninsular Malaysia. The Medical Journal of Malaysia.

[3] Cobo-Cuenca AI, Martín-Espinosa NM, Rodríguez-Borrego MA, Carmona-Torres JM (2019). Determinants of satisfaction with life and self-esteem in women with breast cancer. Quality of Life Research: An International Journal of Quality of Life Aspects of Treatment, Care and Rehabilitation.

[4] Robinson JP, Shaver PR, Wrightsman LS (2013). Measures of personality and social psychological attitudes: Measures of social psychological attitudes.

[5] Dini G, Quaresma M, Ferreira L (2001). Adaptação cultural e validação da Versão Brasileira da Escala de auto-estima de Rosenberg. Revista Brasileira de Cirurgia Plástica.

[6] Kobayashi M, Ohno T, Noguchi W, Matsuda A, Matsushima E, Kato S, Tsujii H (2009). Psychological distress and quality of life in cervical cancer survivors after radiotherapy: Do treatment modalities, disease stage, and self-esteem influence outcomes?. International Journal of Gynecologic Cancer.

[7] Li C-C, Chen M-L, Chang T-C, Chou H-H, Chen M-Y (2015). Social support buffers the effect of self-esteem on quality of life of early-stage cervical cancer survivors in Taiwan. European Journal of Oncology Nursing.

[8] Dauchy S, Dolbeault S, Reich M (2013). Depression in cancer patients. EJC Supplements.

[9] Die Trill M (2013). Anxiety and sleep disorders in cancer patients. EJC Supplements.

[10] Hernández Blázquez M, Cruzado JA (2016). A longitudinal study on anxiety, depressive and adjustment disorder, suicide ideation and symptoms of emotional distress in patients with cancer undergoing radiotherapy. Journal of Psychosomatic Research.

[11] Mitchell AJ, Chan M, Bhatti H, Halton M, Grassi L, Johansen C, Meader N (2011). Prevalence of depression, anxiety, and adjustment disorder in oncological, haematological, and palliative-care settings: A meta-analysis of 94 interview-based studies. The Lancet Oncology.

[12] NCCN Practice Guidelines for the Management of Psychosocial Distress (1999). National comprehensive cancer network. Oncology (Williston Park).

[13] Satin JR, Linden W, Phillips MJ (2009). Depression as a predictor of disease progression and mortality in cancer patients. Cancer.

[14] Achimas-Cadariu P, Iancu M, Pop F, Vlad C, Irimie A (2015). Psychological screening and health related quality of life in Romanian breast cancer survivors. Journal of Evidence-Based Psychotherapies.

[15] Kolubinski DC, Marino C, Nikčević AV, Spada MM (2019). A metacognitive model of self-esteem. Journal of Affective Disorders.

[16] Kuster F, Orth U, Meier LL (2012). Rumination mediates the prospective effect of low self-esteem on depression: A five-wave longitudinal study. Personality & Social Psychology Bulletin.

[17] Sowislo JF, Orth U (2013). Does low self-esteem predict depression and anxiety? A meta-analysis of longitudinal studies. Psychological Bulletin.

[18] Wang Y, Yi J, He J, Chen G, Li L, Yang Y, Zhu X (2014). Cognitive emotion regulation strategies as predictors of depressive symptoms in women newly diagnosed with breast cancer. Psycho-Oncology.

[19] Aldao A, Nolen-Hoeksema S, Schweizer S (2010). Emotion-regulation strategies across psychopathology: A meta-analytic review. Clinical Psychology Review.

[20] Thompson RA (1994). Emotion regulation: A theme in search of definition. Monographs of the Society for Research in Child Development.

[21] Carpenter JK, Conroy K, Gomez AF, Curren LC, Hofmann SG (2019). The relationship between trait mindfulness and affective symptoms: A meta-analysis of the five facet mindfulness questionnaire (FFMQ). Clinical Psychology Review.

[22] Tomlinson ER, Yousaf O, Vittersø AD, Jones L (2018). Dispositional mindfulness and psychological health: A systematic review. Mindfulness.

[23] Bajaj B, Robins RW, Pande N (2016). Mediating role of self-esteem on the relationship between mindfulness, anxiety, and depression. Personality and Individual Differences.

[24] Pepping CA, O’Donovan A, Zimmer-Gembeck MJ, Hanisch M (2014). Is emotion regulation the process underlying the relationship between low mindfulness and psychosocial distress?. Australian Journal of Psychology.

[25] Coffey KA, Hartman M (2008). Mechanisms of action in the inverse relationship between mindfulness and psychological distress. Complementary Health Practice Review.

[26] Faller H, Schuler M, Richard M, Heckl U, Weis J, Küffner R (2013). Effects of psycho-oncologic interventions on emotional distress and quality of life in adult patients with cancer: Systematic review and meta-analysis. Journal of Clinical Oncology: Official Journal of the American Society of Clinical Oncology.

[27] Kim SH, Kook JR, Kwon M, Son MH, Ahn SD, Kim YH (2015). The effects of laughter therapy on mood state and self-esteem in cancer patients undergoing radiation therapy: A randomized controlled trial. Journal of Alternative and Complementary Medicine (New York).

[28] de Vries M, Stiefel F, Goerling U (2014). Psycho-oncological interventions and psychotherapy in the oncology setting. Psycho-Oncology.

[29] Grégoire, C., Bragard, I., Jerusalem, G., Etienne, A.-M., Coucke, P., Dupuis, G.,… Faymonville, M.-E. (2017). Group interventions to reduce emotional distress and fatigue in breast cancer patients: A 9-month follow-up pragmatic trial. *British Journal of Cancer*, *117*(10), 1442–1449. 10.1038/bjc.2017.326. 10.1038/bjc.2017.326 PMC5680472 28926526

[30] Horneber M, Bueschel G, Dennert G, Less D, Ritter E, Zwahlen M (2012). How many cancer patients use complementary and alternative medicine: A systematic review and metaanalysis. Integrative Cancer Therapies.

[31] John GM, Hershman DL, Falci L, Shi Z, Tsai W-Y, Greenlee H (2016). Complementary and alternative medicine use among US cancer survivors. Journal of Cancer Survivorship: Research and Practice.

[32] Kwekkeboom KL, Cherwin CH, Lee JW, Wanta B (2010). Mind-body treatments for the pain-fatigue-sleep disturbance symptom cluster in persons with cancer. Journal of Pain and Symptom Management.

[33] Vanhaudenhuyse A, Laureys S, Faymonville M-E (2014). Neurophysiology of hypnosis. Neurophysiologie Clinique/Clinical Neurophysiology.

[34] Spiegel D (1991). Neurophysiological correlates of hypnosis and dissociation. The Journal of Neuropsychiatry and Clinical Neurosciences.

[35] Cramer H, Lauche R, Paul A, Langhorst J, Kümmel S, Dobos GJ (2015). Hypnosis in breast cancer care: A systematic review of randomized controlled trials. Integrative Cancer Therapies.

[36] Tellez, A., Rodriguez-Padilla, C., Martinez-Rodriguez, J. L., Juarez-Garcia, D. M., Sanchez-Armass, O., Sanchez, T.,… Jaime-Bernal, L. (2017). Psychological effects of group hypnotherapy on breast cancer patients during chemotherapy. *Journal of Clinical Hypnosis*, *60*(1), 68–84. 10.1080/00029157.2016.1210497. 10.1080/00029157.2016.1210497 28557680

[37] Otani A (2016). Hypnosis and mindfulness: The Twain finally meet. The American Journal of Clinical Hypnosis.

[38] Elkins GR, Roberts RL, Simicich L (2018). Mindful self-hypnosis for self-care: An integrative model and illustrative case example. The American Journal of Clinical Hypnosis.

[39] van der Velden, A. M., Kuyken, W., Wattar, U., Crane, C., Pallesen, K. J., Dahlgaard, J.,… Piet, J. (2015). A systematic review of mechanisms of change in mindfulness-based cognitive therapy in the treatment of recurrent major depressive disorder. *Clinical Psychology Review*, *37*, 26–39. 10.1016/j.cpr.2015.02.001. 10.1016/j.cpr.2015.02.001 25748559

[40] Grégoire C, Faymonville M-E, Vanhaudenhuyse A, Charland-Verville V, Jerusalem G, Willems S, Bragard I (2020). Effects of an intervention combining self-care and self-hypnosis on fatigue and associated symptoms in post-treatment cancer patients: A randomized-controlled trial. Psycho-Oncology.

[41] Grégoire C, Faymonville M-E, Vanhaudenhuyse A, Charland-Verville V, Jerusalem G, Bragard I (2018). Randomized controlled trial of an 8-week intervention combining self-care and hypnosis for post-treatment cancer patients: Study protocol. BMC Cancer.

[42] Polit DF, Gillespie BM (2010). Intention-to-treat in randomized controlled trials: Recommendations for a total trial strategy. Research in Nursing & Health.

[43] Gravel J, Opatrny L, Shapiro S (2007). The intention-to-treat approach in randomized controlled trials: Are authors saying what they do and doing what they say?. Clinical Trials (London, England).

[44] Chang VT, Hwang SS, Feuerman M (2000). Validation of the Edmonton symptom assessment scale. Cancer.

[45] Faymonville M-E, Bejenke C, Hansen E, Cyna AM (2010). Hypnotic techniques. Handbook of communication in anesthesia and critical care.

[46] Vanhaudenhuyse, A., Gillet, A., Malaise, N., Salamun, I., Grosdent, S., Maquet, D.,… Faymonville, M.-E. (2018). Psychological interventions influence patients’ attitudes and beliefs about their chronic pain. *Journal of Traditional and Complementary Medicine*, *8*(2), 296–302. 10.1016/j.jtcme.2016.09.001. 10.1016/j.jtcme.2016.09.001 PMC5934699 29736385

[47] Rosenberg M (1979). Conceiving the self.

[48] Vallieres EF, Vallerand RJ (1990). Traduction et validation canadienne-française de l’échelle de l’estime de soi de Rosenberg. International Journal of Psychology.

[49] Zigmond AS, Snaith RP (1983). The hospital anxiety and depression scale. Acta Psychiatrica Scandinavica.

[50] Baer, R. A., Smith, G. T., Lykins, E., Button, D., Krietemeyer, J., Sauer, S.,… Williams, J. M. G. (2008). Construct validity of the five facet mindfulness questionnaire in meditating and nonmeditating samples. *Assessment*, *15*(3), 329–342. 10.1177/1073191107313003. 10.1177/1073191107313003 18310597

[51] Garnefski N, Kraaij V, Spinhoven P (2001). Negative life events, cognitive emotion regulation and emotional problems. Personality and Individual Differences.

[52] Cohen J (1977). Statistical power analysis for the behavioral sciences.

[53] Carlson LE (2016). Mindfulness-based interventions for coping with cancer. Annals of the New York Academy of Sciences.

[54] Applebaum, A. J., Panjwani, A. A., Buda, K., O’Toole, M. S., Hoyt, M. A., Garcia, A.,… Mennin, D. S. (2018). Emotion regulation therapy for cancer caregivers-an open trial of a mechanism-targeted approach to addressing caregiver distress. *Translational Behavioral Medicine*. 10.1093/tbm/iby104. 10.1093/tbm/iby104 PMC7394488 30395306

[55] Fassbinder E, Schweiger U, Martius D, Brand-de Wilde O, Arntz A (2016). Emotion regulation in schema therapy and dialectical behavior therapy. Frontiers in Psychology.

[56] Berking M, Wupperman P, Reichardt A, Pejic T, Dippel A, Znoj H (2008). Emotion-regulation skills as a treatment target in psychotherapy. Behaviour Research and Therapy.

[57] Medvedev ON, Krägeloh CU, Narayanan A, Siegert RJ (2017). Measuring mindfulness: Applying generalizability theory to distinguish between state and trait. Mindfulness.

[58] Slonim J, Kienhuis M, Di Benedetto M, Reece J (2015). The relationships among self-care, dispositional mindfulness, and psychological distress in medical students. Medical Education Online.

[59] Randal C, Pratt D, Bucci S (2015). Mindfulness and self-esteem: A systematic review. Mindfulness.

[60] Grégoire, C., Nicolas, H., Bragard, I., Delevallez, F., Merckaert, I., Razavi, D.,… Vanhaudenhuyse, A. (2018). Efficacy of a hypnosis-based intervention to improve well-being during cancer: A comparison between prostate and breast cancer patients. *BMC Cancer*, *18*(677), 677. 10.1186/s12885-018-4607-z. 10.1186/s12885-018-4607-z PMC6013950 29929493

